# A pooled long-term follow-up after radiotherapy for prostate cancer with and without a rectal hydrogel spacer: impact of hydrogel on decline in sexual quality of life

**DOI:** 10.3389/fonc.2023.1239104

**Published:** 2023-10-11

**Authors:** Zachary A. Seymour, Michael Pinkawa, Stephanie Daignault-Newton, Walter Bosch, Jeff M. Michalski, Hiram Gay, Daniel A. Hamstra

**Affiliations:** ^1^Department of Radiation Oncology, Beaumont Health, Dearborn, MI, United States; ^2^William Beaumont School of Medicine, Oakland University, Rochester, MI, United States; ^3^Department of Radiation Oncology, Rheinisch-Westfälische Technische Hochschule (RWTH) Aachen University, Aachen, Germany; ^4^Department of Radiation Oncology, Robert Janker Klinik, Bonn, Germany; ^5^Department of Biostatistics, Michigan Medicine, Ann Arbor, MI, United States; ^6^Department of Radiation Oncology and School of Medicine, Washington University, St. Louis, MO, United States; ^7^Department of Radiation Oncology, Baylor College of Medicine, Houston, TX, United States

**Keywords:** prostate, radiation, quality of life, sexual function, rectal spacer

## Abstract

**Purpose:**

The purpose of this study was to analyze the impact of prostate rectal spacers on sexual quality of life (QOL) following external beam radiation therapy (RT).

**Methods and materials:**

Patient- reported QOL was evaluated using the Expanded Prostate Cancer Index Composite (EPIC). Patients were pooled from two sources: a randomized controlled trial and a non-randomized cohort of patients from a single institution. Both cohorts used the same spacing product and QOL instrument. Analysis was limited to those with good baseline pre-treatment sexual QOL (EPIC >/= 60). Differences in QOL summary score and individual items were assessed compared with baseline and between treatment arms.

**Results:**

A total of 128 men had good baseline sexual function and were evaluated (64% with spacer and 36% without) with QOL data available for median 33 months (range: 2.5–69.4 months). Men without spacer were more likely to have declines in sexual function (*p* < 0.0001), bother (*p* = 0.0002), and sexual summary score (*p* < 0.0001). A minimally important difference of 10 points (1xMID) and 20 point (2xMID) was more likely without rectal spacer [10 points: odds ratio 3.53, (95% confidence interval 1.11–11.2), *p* = 0.032; 20 points: odds ratio 3.29, (95% confidence interval 1.16–9.33), *p* = 0.025]. Seven of 13 QOL items were statistically superior with hydrogel (six of nine functional and one of four bother), while no items were statistically superior for control. At baseline, more men treated with hydrogel had erections sufficient for intercourse; however, when analyzed only by the men with best baseline erectile potential and excluding those with worse function, the benefit of rectal spacing was maintained with a higher likelihood of preservation of erections sufficient for intercourse in those treated with hydrogel.

**Conclusion:**

In this pooled analysis of QOL after prostate RT, the utilization of a hydrogel spacer was associated with better sexual QOL, less men with measurable declines in sexual QOL, and higher rates of adequate erectile function.

## Highlights

- Use of a prostate rectal spacer during prostate RT has been shown to decrease dose to the penile bulb.
- In this study men from 2 different cohorts were evaluated for sexual quality of life before and after RT as a function of prostate-rectal spacer.
- The presence of spacer resulted in better preservation of sexual function, less sexual bother, and less decline in potency.

## Introduction

External beam radiation therapy (RT) is commonly utilized to treat men with prostate cancer with long-term cancer-specific outcomes similar for surgery or RT, while patient- reported outcomes (PROs) show greater differences between treatments (1, 2). Following surgery, there is worse urinary incontinence and sexual dysfunction, while there are greater declines in bowel function after RT (1–6). These results were confirmed with two phase 3 trials documenting superior preservation of sexual function with RT- based treatments as compared with radical surgery (4–6). Nerve sparing and robotic surgery have been reported to improve the sexual profile after RP; however, substantial declines in sexual quality of life (QOL) after surgery remain common (3). For RT, sexual declines are common with multiple reports suggesting that the penile bulb (PB) may act as a dosimetric surrogate for RT-associated erectile dysfunction (ED) with the QUANTEC analysis identifying a mean PB dose of 50 Gy as associated with ED (3, 7). Continued gains in image guidance, intensity modulation, and stereotactic RT have allowed for smaller margins and more targeted RT, which may in term lead to better PROs, but to date, there has not been a clear intervention, which has been demonstrated to preserve sexual function following external beam RT (8–11).

Currently, there are two commercially available rectal spacer hydrogels to provide distance between the prostate gland and the rectum (12, 13). Clinical trials demonstrated reduced rectal toxicity and better bowel QOL with hydrogel with either conventionally (12) or moderately hypofractionated RT (13). Somewhat unexpectedly in the SpaceOAR phase 3, trial there was reduced radiation dose to the PB in those treated with hydrogel, which was true for mean dose, maximum dose, and percentage of PB receiving all doses between 10 and 30 Gy (all *p* <.05) (12, 14). As the hydrogel often accumulates more at the base of the prostate relative to apex, it is possible by displacing the prostate further away from the base of the penis and the pudendal neurovascular bundles that the dose to these erectile structures may be spared in an unanticipated manner (15). A similar reduction in PB dose was noted in a previous study using stereotactic prostate RT with reductions in maximal and mean doses to PB with SpaceOAR (both *p* < 0.0001) (16). Furthermore, in the SpaceOAR phase 3 trial for men with erections sufficient for intercourse at baseline increasing mean PB dose directly correlated with worse ED (*p* = .03). Similarly, at 3 years, more men potent at baseline and treated with spacer had “erections sufficient for intercourse “ as compared with men treated without (control 37.5% *vs.* spacer 66.7%, *P* = .046) (14). A similar observation was made in the single institutional SpaceOAR cohort evaluated herein where at the time of the last questionnaire, 24% (with spacer) *versus* 3% (without spacer) reported erections firm enough for intercourse (*P* <.01) (17).

Therefore, presented here is a pooled analysis of these two series of patients focusing on sexual-related QOL: a prospective phase 3 multi-centered randomized trial and a prospective non-randomized single institution analysis of patients sequentially treated where, in each study, men were treated either with or without a hydrogel spacer. In both studies, SpaceOAR (Boston Scientific, Boston, MA, USA) was utilized as the prostate-rectal spacing device. In each study, those treated with hydrogel and RT had numerically superior sexual QOL over time, but these results were of borderline significance, and as a result, a pooled analysis using individual patient data was deemed warranted.

## Materials and methods

### Patient selection and treatment parameters

The details of the phase 3 trial and non-randomized cohort were previously reported, where in each case, men were treated with conventionally fractionated RT using IMRT with or without SpaceOAR (Boston Scientific, Boston, MA) (12, 17). On the phase 3 trial, men with National Comprehensive Cancer Network–determined low- or intermediate-risk prostate cancer and a Zubrod performance status of 0 –1 were enrolled in a multi-institutional institutional review board–approved single-blind phase 3 trial (Clinical Trials ID: NCT01538628) from 20 separate institutions. The exclusion criteria included prostate volume ≥ 80 cm^3^, extra prostatic extension, > 50% positive biopsy cores, previous or planned use of ADT, and/or previous treatment of prostate cancer. The patients were randomized 2:1 to the hydrogel or control group, with all men receiving fiducial markers for IGRT. The patients were unaware of the treatment allocation and had the fiducial markers or markers plus the hydrogel placed without knowing to which treatment they were randomized. Magnetic resonance imaging (MRI)–based planning was used, with the post-fiducial marker computed tomography (CT) scan fused with the MRI. The RT plans were evaluated by an independent core laboratory before treatment for compliance to the protocol guidelines and determination of the dosimetric endpoints. There were pre-specified goals for rectal and urinary planning targets but no protocol specified constraints for PB. The clinical target volume (CTV) was the prostate with or without the seminal vesicles at the physician’s discretion. A planning target volume (PTV) margin of 5 mm–10 mm was used. The radiation dose was 79.2 Gy in 1.8-Gy daily fractions, delivered 5 days weekly. CT- based daily image guidance was utilized for treatment delivery with alignment to the fiducials.

In the non-randomized cohort, all 114 patients were treated from 2010 to 2011 with external beam RT to the prostate without pelvic lymph nodes. Treatment plans were based on a CT scan in the supine position with a full bladder, within 3 –5 days after hydrogel injection. Additionally, T2-weighted MRI scans were performed with image fusion in the first 27 patients and then CT scans alone were used thereafter. For the PTV, 8-mm lateral and anterior, 5-mm superior and inferior, and 4-mm posterior margins were added to the CTV (corresponding to prostate with or without seminal vesicles) contours. Treatment was performed with a five-field intensity-modulated RT to a total dose of 76 Gy (*n* = 96) or 78 Gy (*n* = 18, all with hydrogel). The same objectives and constraints were used for inverse intensity modulated RT treatment planning for all patients. Ultrasound-based image guidance was applied before each fraction.

No patient in either data set was treated with androgen deprivation therapy.

### Quality of life data

Patient- reported QOL was obtained prior to RT and in follow- up with the Expanded Prostate Cancer Index Composite short form (EPIC-26) (1). Practice patterns of follow-up varied by each cohort. In the prospective randomized study, follow- up occurred every 3 months for 2 years and then every 6 months. The non-randomized cohort obtained patient- reported QOL surveys prior to treatment, at the completion of RT, and at approximately 2, 17, and 63 months after RT.

Overall, a total of 380 men treated had baseline EPIC evaluated. This includes 245 patients treated with and 135 treated without rectal spacer. Analysis was limited to patients with good baseline sexual function by baseline EPIC sexual domain score (>/= 60).

### Statistical analysis

Patients without hydrogel spacer were labeled as “ control,” while those treated with hydrogel were labeled “hydrogel.” Baseline characteristics between groups were compared with a Wilcoxon rank-sum test. The overall EPIC domain sexual score, as well as validated subsets of scores evaluating sexual function and sexual bother were assessed over time using linear mixed models to account for within patient correlation due to repeated measures. A previously defined threshold for MID to define clinical significance was utilized as a change of 10 points in the sexual summary score, while a “ severe” score change of 20 points was considered a MIDx2 (18). Responses at the 24-month or later response time for each sexual domain question were presented by dichotomizing the responses and presenting the proportion in each group with the associated chi-square test. In patients with more than one QOL assessment at 24 months or later, the latest follow-up was utilized for the assessment of QOL at last contact.

For the overall sexual QOL summary score, sexual function, and sexual bother sub-domains, the changes in the EPIC scores from baseline were evaluated in linear mixed models with the fixed effects of presence of rectal hydrogel spacer, time of questionnaire completion since treatment, baseline sexual score, cohort, and the interaction of presence of rectal hydrogel spacer and questionnaire completion time. Repeated measures within a patient were accounted for using an autoregressive correlation structure. Pairwise testing to compare the hydrogel spacer groups at different times was done with contrasts within the modeling framework. Each binary MID variable was presented with the proportion of patients in each hydrogel spacer group at each time with an MID or MID2x, respectively. Separate logistic models for MID and MID2x was used to estimate the odds of an MID (or MID2x) between the hydrogel spacer groups at the last questionnaire time adjusting for cohort. Analysis was performed in SAS, version 9.4 (SAS Institute, Cary, NC). A 5% types I error rate was used to determine statistical significance.

## Results

### Patient baseline characteristics

A total of 128 men (33.7% of the cohort) were deemed suitable for analysis of sexual function as they had at least adequate baseline sexual QOL (as defined by an EPIC sexual summary score of at least 60). Clinical characteristics were similar between patients with or without rectal spacer (**Table 1**). This included 64.8% (*n* = 83) with hydrogel and 35.2% (*n* = 45) without hydrogel. Median follow-up was 33 months (range: 2.5–69.4 months) with QOL available for 69 men beyond 24 months. There was no difference in duration of follow-up between those with or without rectal spacer (**Table 1**). In this group with better baseline sexual function, those treated with hydrogel at the start of treatment had higher sexual function (70.2 *vs.* 66.4, *p* = 0.045), less issue with sexual bother (92.6 *vs.* 88.2, *p* = 0.012), and higher sexual summary scores (77.2 *vs.* 73.3, *p* = .026); although the difference between groups in summary score (3.9 points) was below the MID of 10–12 points. Furthermore, the difference in each case was less than one half the standard deviation (**Supplementary Table S1**) indicating no clinically significant differences in sexual function, bother, or overall sexual summary score between the two groups of men at baseline.

**Table 1:** Pre- treatment patient characteristics with good baseline sexual function by treatment group.

	Hydrogel (*N* = 83)	Control (*N* = 45)	*p*-value
**Age** **Mean (StdDev)**	64.2 (7.4)	65.9 (6.7)	0.20
**Percent positive cores Median (IQR)**	16.7% (14.3% –33.3%)	16.7% (12.5%– 34.6%)	0.76
**Baseline PSA ng/mL Median (Min –Max)**	6.7 (5 – 9.2)	6.3 (4.5– 9.2)	0.96
**Prostate volume median (Min –Max)**	51.3 (43.0– 62.4)	57.5 (49.0– 69.5)	0.15
**Follow- up (months)** **[Median (IQR)]**	33.0 (15.0–38.8)	35.2 (16.0–38.9)	0.26
**Sexual function domain [Mean (StdDev)]**	70.2 (10.8)	66.4 (8.8)	0.045
**Sexual bother domain** **[Mean (StdDev)]**	92.6 (9.6)	88.2 (8.8)	0.012
**Sexual summary score** **[Mean (StdDev)]**	77.2 (8.5)	73.3 (10.8)	0.026

### Patient- reported sexual quality of life

**Figure 1:**
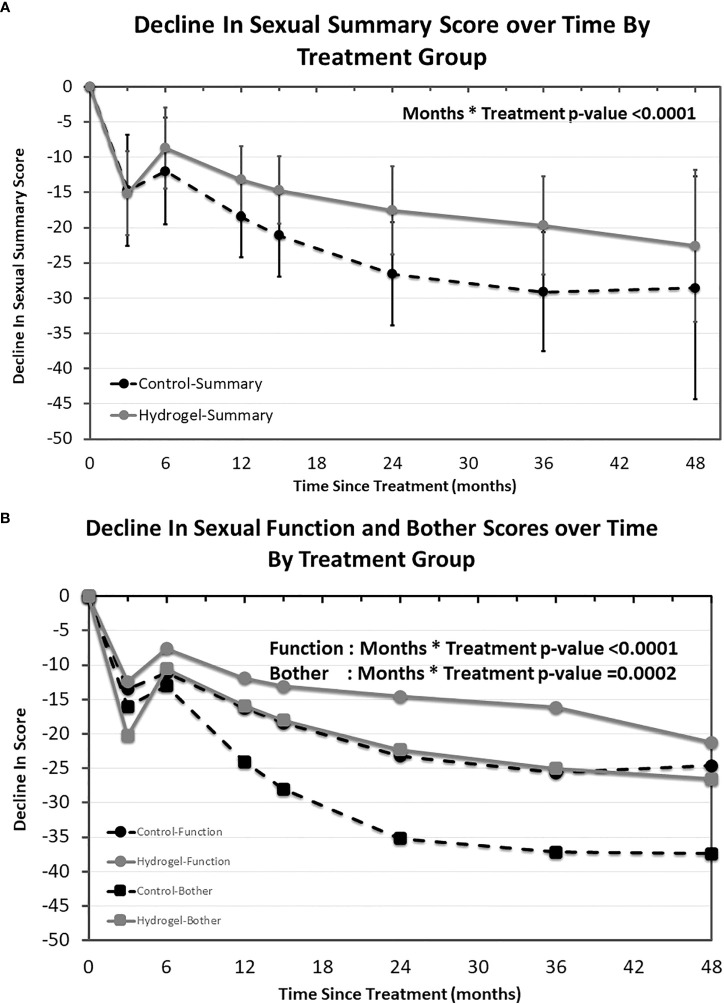
EPIC sexual quality of life over time by treatment group. Modeled mean EPIC sexual quality of life summary scores and 95% confidence interval by treatment group **(A)** as well as EPIC sexual function and sexual bother sub-domains **(B)**.

Declines in EPIC domain scores were assessed after controlling for the presence of hydrogel spacer, time, treating cohort, and the interaction between rectal hydrogel spacer and time. The EPIC sexual summary score declined over time following RT both in men treated with or without hydrogel (**Figure 1A**). The mean estimates of change in summary score were statistically different over time between treatment groups with significantly smaller declines in those treated with hydrogel as compared with those treated without rectal spacing (months * treatment *p* < 0.0001). Overall sexual scores at 36 months declined by 29.1 points in the control group, which was greater than the MIDx2 (20-point decline), while the decline in men treated with hydrogel was substantially lower (−19.7 points), a difference of 9.4 points favoring the hydrogel arm. The differences between arms favoring hydrogel at 12, 24, 36, and 48 months were 5.3, 9.1, 9.4, and 6.0 points, respectively. These differences were less than the established 10-point MID threshold but, in each case, was greater than one half the baseline standard deviation in this data set (4.1 points).

The validated EPIC sexual function and bother sub-domains were also evaluated over time (**Figure 1B**). Both sexual function and sexual bother scores decreased over time and in each case smaller declines were observed in the hydrogel spacer treated patients (sexual function sub-domain months * treatment *p* = < 0.0001 and sexual bother sub-domain months * treatment *p* = 0.002). Similar to the sexual summary scores, both function and bother began to separate from 6 months onward in all cases favoring men treated with hydrogel with differences at 12, 24, 36, and 48 months of 4.4, 8.7, 9.5, and 3.4 points, respectively, for function (where one half the standard deviation was 5.2 points) and 8.1, 12.9, 12.2, and 10.9 points, respectively, for bother (where one half the standard deviation was 5.1 points).

**Figure 2:**
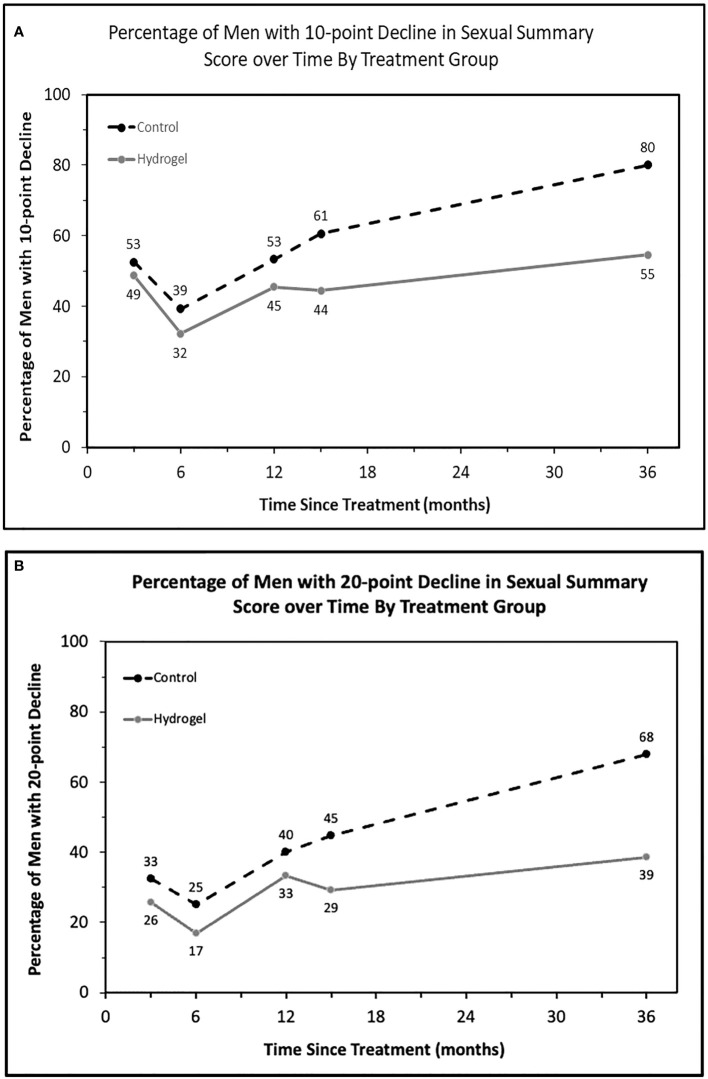
The proportion of patients with defined thresholds of decline in sexual summary score over time by treatment group. **(A)** Minimally important decline (10 points, MIDx1) in sexual summary score by treatment and **(B)** twice the MID (20 points).

In addition to assessing average declines for each treatment group, we next assessed the proportion of men meeting the minimally important decline (MID) in sexual function over time. For this analysis, we utilized cut points of 10 points (**Figure 2A**) as well as 20 points (**Figure 2B**) as a measure of severe decline. As seen for average scores, there was an initially greater impact on sexual QOL at both 10- and 20-point thresholds observed at 3 months in both men treated with and without hydrogel with modest improvements noted at 6 months followed by continued declines in sexual QOL over time. From 6 months onward, a smaller portion of men had measurable declines in sexual QOL when treated with hydrogel. At last follow-up, men without spacer were more than 3 times as likely to have a decline in EPIC QOL meeting the 10- or 20-point thresholds: 10-point decline (MIDx1), odds ratio 3.53, (95% confidence interval 1.11–11.2), *p* = 0.032 as well as 20-point decline (MIDx2), odds ratio 3.29, (95% confidence interval 1.16–9.33; *p* = 0.025).

**Figure 3:**
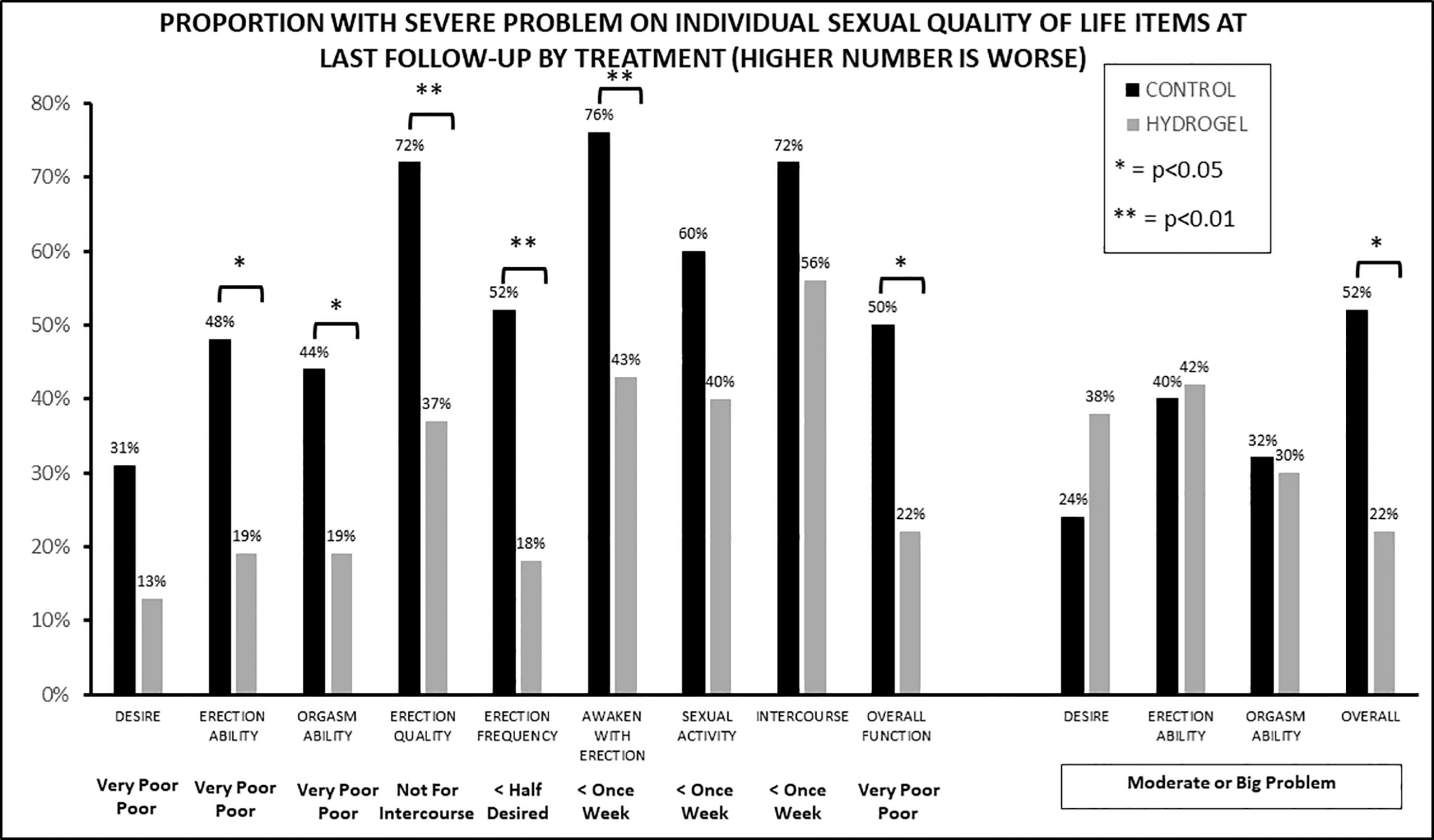
The proportion of patients with severe impact on sexual function (nine items) or bother (four items) at last follow-up by treatment group.

To assess which aspects of sexual QOL were impacted, we next evaluated the sexual domain by assessing which percentage of patients had a substantial impact on QOL at last follow-up for each of the 13 items making up the EPIC sexual QOL instrument. For example, for function men were stratified by “poor or very-poor erectile function” as compared with a lesser impact or for bother by those with a “moderate or big problem” as compared with lower degrees of bother (**Figure 3**). This revealed numerically better QOL with hydrogel for 11 of 13 items (nine of nine functions and two of four bothers) with seven of 13 items having statistically significant differences favoring hydrogel (six of nine functions and one of four bothers). In contrast, no items in the functional domain favored the control group either numerically or statistically, while two of four items in the bother domain favored the control group (namely, bother from sexual desire and bother from erection ability), but neither of these were statistically significant. Looking at the single-item assessors of sexual QOL, overall sexual function favored hydrogel with 22% reporting “poor or very poor function” when treated with hydrogel as compared with 50% treated without hydrogel (*p* = 0.016). Similarly, overall sexual bother favored men treated with hydrogel where 22% of men reported a “moderate or big problem” with bother as compared with 52% in the control group (*p* = 0.011).

**Figure 4:**
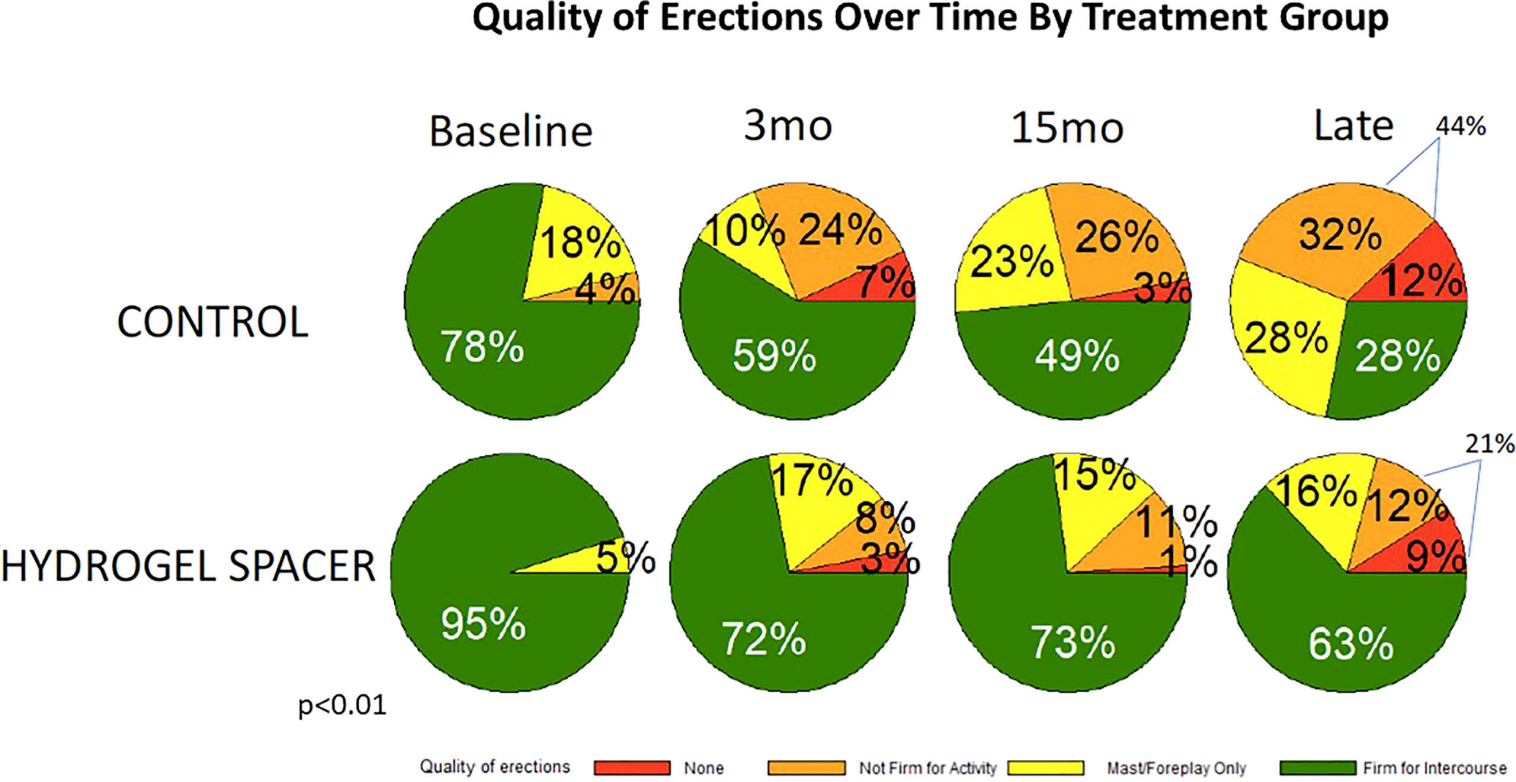
The quality of erections at baseline and over time by treatment group.

Finally, the single EPIC item on “erections sufficient for intercourse” has previously been assessed as an evaluation of sexual QOL (**Figure 4**) (3). Of note, there were differences between those treated with and without hydrogel at baseline with 95% (experimental) and 78% (control) with erections sufficient for intercourse at baseline. Even taking this difference into account, declines in erectile ability were greater in those treated with RT without rectal spacing. For control men, if they had “erections sufficient for intercourse” at baseline 36% maintained this at last follow-up while for hydrogel patients in those with “erections sufficient for intercourse” at baseline 66% maintained this at last follow-up.

In men with hydrogel at late follow- up, 63% of patients had erections firm enough for intercourse and 21% had erectile function not firm enough for any activity or no erections, while in those treated without hydrogel, 28% had adequate erectile function for intercourse and 44% without any erectile function firm enough for any activity or no erections. This difference in erections sufficient for intercourse at last follow-up (35%) was more than twice the difference at baseline (17%).

## Discussion

The impact of prostate cancer treatment on sexual function has a substantial influence upon men’s decisions in regard to treatment and upon both their QOL and regret following treatment (19–21). In men that do have adequate erectile function who are treated with RT, we still lack clear comprehensive data for all potential organs at risk, which lead to sexual decline after RT (7, 22). The PB has most frequently been used as a metric to assess RT plan quality and as a surrogate for the impact on sexual QOL. The PB, although it may not be a primary organ for erectile function, serves as a surrogate for other adjacent but incompletely understood organs at risk such the peudenal arteries or corporal bodies.

In this context, the QUANTEC analysis of the PB is the closest accepted norm to minimize risk of erectile function decline, and it concluded that mean doses of 50 Gy to the PB were associated with ED (7). Nevertheless, contemporary studies point to substantially lower RT doses still impacting ED. For instance an analysis of the UK CHHiP trial identified mean PB doses of <26 Gy in 2 Gy fractions as a target to optimize preservation of sexual function following RT (23), while a previous analysis of the SpaceOAR phase 3 trial noted improvements in PB dosimetry in those treated with SpaceOAR including reduced doses across the full range assessed (12, 14). Despite this, in the overall sample, this did not lead to differences in sexual QOL; however, two-thirds of the men had very poor sexual function as defined by an EPIC sexual summary score < 60 at baseline and consequently were not suitable for assessment of sexual QOL.

As a result, the pooled analysis reported here was undertaken which increased the number of potent men for analysis by 45% from 88 to 128. The results confirmed those observed previously with statistically significant differences in sexual function (*p* < 0.0001), bother (*p* = 0.0002), and summary scores (*p* < 0.0001) over time between treatment arms, which all favored those treated with hydrogel. The impacts were greatest on function (six of nine items impacted) as compared with bother (one of four items impacted). In simpler terms, men who were potent at baseline and treated with RT and hydrogel were nearly twice as likely to have an erection sufficient for intercourse at last follow-up than control men (63% *vs.* 28%). Nevertheless, even with hydrogel, declines in sexual function were common after RT with men approaching an MID at 6 months after RT on average and at late follow-up almost double that amount of decline. However, consistent with previous reports, men without hydrogel faired far worse in regard to sexual function.

Interpreting the clinical significance of QOL scores is often debated with efforts taken to separate statistically significant but meaningless differences from those which are both statistically different and clinically relevant (1, 24). To that end, we utilized the established MID value of 10–12 points published by Skolarus et al. for the EPIC instrument as the threshold for analysis (18). This MID value was developed based upon both distribution and anchor-based approaches in the PROST-QA data set where for sexual summary score 10 points equated to one-third standard deviation and 12 points one half standard deviation. However, in our data set, particularly when limited to men with better overall sexual QOL (EPIC >= 60), there was a much smaller spread of data, because all men with baseline scores below 60 were excluded and, correspondingly, we observed a much smaller standard deviation of 8.2 points. Within this context, although differences with hydrogel did not meet the previously published MID threshold of 10 points (which corresponded to one half standard deviation in the derivation set used by Skolarus et al.), the differences we observed of 9.1–9.4 points at 24 –36 months were equivalent to the standard deviation in this data set (8.2 points) and indeed twice the one half the standard deviation that is often used to determine clinical significance in QOL data sets (24). Therefore, based upon the threshold of one half standard deviation within this data set, the differences between arms met this threshold for all time points from 12 months onward favoring better sexual QOL in those treated with hydrogel. Nevertheless, men treated with RT and hydrogel continued to have significant and clinically meaningful declines over time, which underlie the needs for additional studies aimed at actively reducing dose to anatomical structures related to sexual decline after RT. In addition, a detailed analysis of potential structures relevant for sexual decline following RT is critically needed (7, 22).

Two single- arm prospective studies on vessel sparing RT appeared to provide improvements in potency and sexual QOL preservation as compared with historical controls (25, 26). In contrast, a recent phase 3 trial of IMRT to spare the PB and corporal bodies did not find a difference in potency sparing at 2 years as measured using the international index of erectile function (IIEF) (47.9% experimental *vs.* 46% control) (27). Of note, this phase 3 trial did not report full dosimetry for the target structures but did note small dosimetric differences for their primary doismetric constraint (PB D90 <=15 Gy) between arms, which was achieved in 88% of experimental patients as compared with 75% of control patients. Therefore, it is not clear from this study that PB sparing does not provide a benefit, but, instead, it may be that in the trial the minimal differences in dosing achieved between arms were not able to demonstrate improvements in sexual function. In comparison, on the SpaceOAR phase 3 trial, the presence of hydrogel reduced the dose across the full range evaluated and was statistically significant for the V10-V30, mean dose, and max dose to the PB. In addition, a greater proportion of men achieved multiple difference dosimetric thresholds that were identified by the CHHiP trial as being associated with erectile function; for instance, 73% of men achieved a Dmax < 58 Gy with SpaceOAR as compared with 53% without. Furthermore, preservation of erectile function was greater in those with lower radiation dose to the PB; for instance, 46% retained potency with Dmax < 58 Gy as compared with 32% with Dmax >= to 58 Gy. Therefore, it is possible that the physical distance provided between the prostate and rectum by the hydrogel also allowed unanticipated dosimetric sparing of the PB with associated improvement in sexual function. As such, we await results of additional prospective trials to further clarify the potential benefit of erectile function sparing RT such as the on-going POTEN-C phase 2 trial, which is assessing neurovascular sparing for ED avoidance using stereotactic RT with a prostate-rectal spacer to help facilitate this process (28).

Limitation of this analysis include (1) not all patients were randomized to treatment with one-third of patients treated as part of a non-randomized cohort. (2) At baseline, there were slight numeric differences in sexual QOL favoring the hydrogel group, but although statistically significant, the differences did not meet the MID threshold as determined by Skolarus et al. (10 points) or even one half standard deviation in this data set. (3) It is possible that those with lower baseline sexual QOL were at greater risk of further declines, although previous analyses have not clearly demonstrated this association. In fact, prior reports have noted that those with lower baseline QOL have smaller net declines, while those with higher baseline QOL have a greater susceptibility (and more to lose) and have been noted to have larger declines. (4) A lack of dosimetric data identifying a mechanistic cause for better preservation of sexual QOL in those treated with RT and hydrogel. (5) All patients in this data set were treated with conventionally fractionated RT of 1.8–2.0 Gy and, as such, it is not clear how this would apply to the presently more commonly utilized moderate or extreme hypofractionation. Nevertheless, the strengths include the prospective evaluation of sexual QOL using the same validated QOL instrument in two independent cohorts. In this pooled analysis with a larger sample size, we observed statistically significant and clinically meaningful differences in sexual QOL throughout the follow-up period when using hydrogel followed by prostate RT compared with RT alone.

## Data availability statement

The data analyzed in this study is subject to the following licenses/restrictions: Date from an industry-sponsored phase 3 trial that are not currently publicly available. Requests to access these datasets should be directed to Daniel.Hamstra@bcm.edu.

## Ethics statement

The requirement of ethical approval was waived by Beaumont Health/Oakland University for the studies involving humans because No identifying information. Only anonymous data used. The studies were conducted in accordance with the local legislation and institutional requirements. The ethics committee/institutional review board also waived the requirement of written informed consent for participation from the participants or the participants’ legal guardians/next of kin because No identifying information. Only anonymous data used.

## Author contributions

Study Design: ZS and DH. Providing Data or Patient Resources: DH, MP, WB, JM, and HG. Data Analysis: SD-N and DH. Drafting of Manuscript: All. Final Manuscript Approval: DH.
