# Supplementary material for: A pooled long-term follow-up after radiotherapy for prostate cancer with and without a rectal hydrogel spacer: impact of hydrogel on decline in sexual quality of life
Source: Front Oncol. 2023 Oct 11;13:1239104. doi: 10.3389/fonc.2023.1239104 (PMC10599244; doi:10.3389/fonc.2023.1239104)
Supplement: Supplementary file 1 [file Table_1.docx]

Supplemental Table 1

Baseline QOL data for cohort with EPIC Summary Score of 60 or more

|  | **Number** | **Label** | **N** | **Mean** | **Std Dev** |
| --- | --- | --- | --- | --- | --- |
| **Baseline** | **128** | \| **Sexual Summary** \| \| --- \| \| **Sexual Function** \| \| **Sexual Bother** \| | \| 128 \| \| --- \| \| 126 \| \| 128 \| | \| 75.9 \| \| --- \| \| 68.8 \| \| 91.1 \| | \| 8.2 \| \| --- \| \| 10.3 \| \| 10.2 \| |
